# Comparison of Baerveldt 101-350 Glaucoma Implant (BGI) versus Trabeculectomy with ExPress™ Shunt for the Treatment of Primary Open Angle Glaucoma

**DOI:** 10.3390/vision1020015

**Published:** 2017-06-07

**Authors:** Anand Bhatt, Zackery Oakey, Hannah Muniz-Castro, Sameh Mosaed

**Affiliations:** 1 Gavin Herbert Eye Institute, Irvine, CA 92697,USA; 2 Irvine School of Medicine, University of California, Irvine, CA 92697, USA

**Keywords:** primary open angle glaucoma, baerveldt glaucoma implant, trabeculectomy, ExPress shunt

## Abstract

*Importance*: Trabeculectomy is very effective in lowering intraocular pressure for the treatment of glaucoma, but it carries with it possible complications and failure. The ExPress shunt (Alcon Laboratories, Fort Worth, TX, USA) is an adjunctive device that can be used at the time of trabeculectomy to create an external fistuliztion. An alternative established and highly efficacious technique is the implantation of a glaucoma drainage device for sustained intraocular pressure (IOP) lowering. Specifically, evidence has established the Baerveldt 101-350 glaucoma implant (BGI) to have the best sustained IOP lowering in long-term follow-up amongst the many options for glaucoma drainage devices. *Objective*: To compare outcomes in eyes that underwent Baerveldt 101-350 glaucoma implant (BGI) and trabeculectomy with ExPress shunt (Trab) in primary open angle glaucoma without any prior incisional glaucoma surgery. *Design*, *Setting*, *and* *Participants*: This was a retrospective study of outcomes in patients identified by CPT codes as having undergone glaucoma implantation or trabeculectomy (with ExPress shunt) for the treatment of Primary Open Angle Glaucoma between 2012 and 2015 at a single institution by 2 fellowship trained glaucoma surgeons. A total of 57 eyes that underwent Baerveldt 101-350 glaucoma implant and 38 eyes that underwent trabeculectomy cases with ExPress™ shunt were included in the study. All patients were diagnosed with primary open angle glaucoma. Cases were included into the study if the patient underwent BGI or trabeculectomy with ExPress shunt without any prior incisional glaucoma surgery. *Main Outcomes and Measures:* Main outcomes included IOP, medications, visual acuity (VA), and secondary glaucoma surgery, if any. Results: Survival rate at 12 months was 85% in the BGI group and 80% in trabeculectomy with ExPress Shunt. A statistically significant difference was not found in the survival distributions between surgery groups using the log–rank test. A total of 12 trabeculectomy and 9 BGI cases failed by our definition of success. These cases were included in the analysis of IOP, number of glaucoma medications, and VA. The mean IOP was reduced from 20.6 ± 5.6 mmHg to 12.4 ± 3.2 mmHg and from 20.7 ± 5.5 mmHg to 11.3 ± 4.8 mmHg at one year post-operation in the BGI group and the trabeculectomy with ExPress shunt group, respectively. On average, the BGI group showed an IOP reduction of 7.7 ± 6.1 mmHg, while trabeculectomy with ExPress shunt experienced a decrease of 7.9 ± 5.2 mmHg at one year post-operation. Medications were reduced from 3.5 ± 0.8 to 2.6 ± 1.3 at one year in the BGI group and from 3.7 ± 0.5 to 0.6 ± 1.8 in the trabeculectomy with ExPress shunt group. At one year post-operation, the BGI group had an average of 0.9 ± 1.1 medication reduction, while trabeculectomy with ExPress shunt cases had a reduction of 3.2 ± 1.3 medications. VA was compared in logMar. At baseline, the average for BGI logMar was 0.5 ± 0.7 and the average for trabeculectomy was 0.2 ± 0.3. At one year post-operation, the BGI group’s VA was 0.4 ± 0.4 while the trabeculectomy with ExPress shunt group’s VA was 0.1 ± 0.1. *Conclusions and Relevance:* The Baerveldt 101-350 glaucoma implant and trabeculectomy (with ExPress™ shunt) may have similar rates of success in the surgical treatment of primary open angle glaucoma in eyes that are naïve to prior incisional glaucoma surgery, with a higher dependence on topical medications post-operation in patients undergoing Baerveldt glaucoma implantation.

## 1. Introduction

Achieving an intraocular pressure (IOP) in the low teens for advanced glaucomatous disease is potentially accomplished with a variety of techniques. When surgical treatment is necessary, trabeculectomy has served as the established gold standard in lowering intraocular pressure. However, this procedure has its complications, risks, and failures. Furthermore, there is a routinely demanding post-operative course for patients with very frequent steroid drops, suture lysis, anti-metabolite injection, and frequent follow-up. For some patients, the practical limitations to trabeculectomy preclude undertaking this surgical choice. Since the introduction of trabeculectomy as a surgical technique, many advancements have taken place to improve post-operative outcomes including the precise titration of anti-metabolites and the use of the ExPress shunt for controlled fistulization.

An alternative established and highly efficacious technique is the implantation of a glaucoma drainage device for sustained IOP lowering. In a recent five-year treatment comparison study of glaucoma implants by Budenz et al. [1], the Baerveldt 101-350 glaucoma implant (BGI) was shown to have the best sustained IOP lowering in long-term follow-up [1,2].

Most glaucoma surgeons have traditionally considered glaucoma drainage devices to be more appropriate for treatment in refractory glaucoma. Recently, groundbreaking studies [3,4] examining treatment outcomes in glaucoma implant surgery versus trabeculectomy have answered significant questions pertinent to the treatment of refractory glaucoma. The authors [3] established that, in eyes that had undergone previous failed trabeculectomy or cataract surgery, Baerveldt 101-350 glaucoma implantation was similarly efficacious to a trabeculectomy in long-term follow-up with fewer potential complications and vision loss in the Baerveldt glaucoma implant group [3]. However, as much as this landmark study contributed to the surgical practice of glaucoma, many questions including methodologies and inclusion/exclusion criteria were left unanswered. At the time the study was performed, the inclusion criteria consisted of eyes that had refractory glaucoma and previous failed trabeculectomy (30%), underwent previous cataract surgery (55%), or both (12%) [3]. Eyes that were naïve to previous incisional surgery were excluded. The use of mitomycin C during trabeculectomy in the TVT trial was standardized to 4 min of exposure resulting in a higher rate of hypotony than previously reported in the literature. The patients included in the TVT study consisted of a broader variety of glaucoma including angle closure, neovascular glaucoma, and uveitic glaucoma, which are considered very different diseases than the more common variety of primary open angle glaucoma. At the time of the TVT trial, the ExPress device was not commonly used as an adjunctive device to trabeculectomy, and is now a commonly used device with evidence supporting fewer post-operative complications and quicker visual recovery compared to standard trabeculectomy [4]. Given the multi-center trial nature of TVT and the subjective post-operative management of trabeculectomy amongst many surgeons, the outcomes can vary depending on the surgeons’ preferences in performing laser suture lysis and steroid regimen.

The present study reports outcomes in eyes that underwent each of these surgical modalities with open angle glaucoma, and which are naïve to previous incisional glaucoma surgery. Further investigations are needed to determine if a glaucoma drainage device can offer equally efficacious IOP lowering results as trabeculectomy as a primary surgical treatment in eyes with open angle glaucoma.

## 2. Methods

### 2.1. Patients and Collection Data

This was a retrospective review of outcomes in patients identified by CPT codes as having undergone glaucoma implantation or trabeculectomy (with ExPress shunt) between 2012 and 2015 at a single institution by two fellowship trained glaucoma surgeons. Institutional review board approval was obtained at UC Irvine prior to gathering data and in accordance with the declaration of Helsink. Fifty-eight eyes underwent Baerveldt 101-350 glaucoma implant surgery, and 38 patients underwent trabeculectomy (with ExPress™, Alcon, Ft. Worth, TX, USA). Demographics of patients undergoing BGI or trabeculectomy procedures are listed in Table 1. Patients excluded from the review had prior incisional glaucoma surgery, non-primary open angle varieties of glaucoma, or less than 1 year of follow-up. Prior laser trabeculoplasty, cataract surgery or age was not exclusion criteria.

Pre-operative data including intraocular pressure (IOP), number of medications, and vision were recorded, and then tracked at 6 weeks, 3 months, 6 months, and 12 months post-operation. Success was defined as achieving at least a 20% reduction in IOP from baseline and maintaining an IOP < 21 at all post-operative follow-up. Complications such as loss in vision and need for re-operation were recorded in follow-up as well. Interventions in the post-operative period such as suture lysis, bleb needling, and subconjunctival injection of anti-metabolite were not recorded as re-operation or complications.

### 2.2. Statistical Analysis

Patients and treatment characteristics in this cohort were described previously. Kaplan–Meier analysis was performed with success defined as IOP ≤ 21 mmHg and at least 20% IOP reduction from baseline for any two consecutive visits after 3 months and no secondary glaucoma surgery. A log–rank test was used to compare survival distributions between surgery groups. Mann–Whitney U and chi-square tests were used to compare continuous and categorical variables between surgery groups. Bonferroni was used to correct for multiple comparisons. All statistical analysis was performed with SAS software (version 9.3; SAS Institute, Inc., Cary, NC, USA).

### 2.3. Surgical Techniques

Both surgeons in this review used identical standardized techniques. The preferred technique for anesthesia was retrobulbar block consisting of 2.5–3.0 cc of a 50:50 mixture of 4% lidocaine and 0.75% bupivacaine. In the patients undergoing trabeculectomy, a fornix-based conjunctival peritomy was performed, followed by a sub-Tenon dissection posteriorly and an application of 0.4 mg/mL mitomycin C applied to sclera by pre-formed weck cell pledgets provided in the Mitosol™ kit (Mobius Therapeutics, St. Louis, MO, USA). These pledgets were applied to sclera posterior to the site of flap creation and left in place for 90 s in each case, followed by vigorous rinsing and removal of residual anti-metabolite with a balanced saline solution. A 3.5 mm × 3 mm triangular scleral flap was then created with the base at the surgical limbus. Beneath this flap, an ExPress Model P50™ (Alcon, Ft. Worth, TX, USA) shunt was used to create a fistula. This flap was then closed with 10-0 nylon sutures in an interrupted fashion (generally with one suture at the apex, and one at each base of the flap) to create a watertight closure. The conjunctival peritomy was then closed with a 10-0 vicryl suture in a running fashion. The surgeons choose to perform laser suture lysis and injection of 5-Fluorouracil subconjunctivally during the post-operative period depending on the examination of the bleb and the IOP at each of the post-operative visits. Post-operatively, all patients were started on topical moxifloxacin for one week and prednisolone acetate 1% every hour while awake and tapered slowly based on examination of the bleb, IOP, and conjunctival hyperemia. Most patients were slowly tapered over 4–6 months.

### 2.4. Glaucoma Implant Surgery

For glaucoma implant surgery, the patients received a Baerveldt 101-350™ implant (Abbot Medical Optics, Santa Ana, CA, USA). The superotemporal quadrant was preferred for placement of the implant, as these patients had no prior implanted device. The initial incision was a 3–4 mm limbal based conjunctival wound, followed by a posterior sub-Tenon dissection to expose and hook the superior and lateral recti muscles with a muscle hook. The implant was then positioned with each of the lateral wings tucked beneath the muscle insertions, and the islets were then sutured and secured to sclera with an 8–0 nylon suture. An 8–0 vicryl was then used to ligate the tube in a water tight fashion. After creation of a paracentesis, anterior dissection was performed at the superior limbus beneath conjunctiva to expose sclera. A 23 gauge needle was used to form a scleral tunnel starting 1.5–2.0 mm behind the limbus at the 12:00 position to minimize the risk of exposure due to eyelid movement. The tube was trimmed with an anterior bevel and introduced into the anterior chamber through this tunnel. The entrance of the tube into this tunnel was then covered with a patch graft and the conjunctival wound closed with an 8–0 vicryl suture in a running fashion. These patients were managed with topical moxifloxacin for one week and prednisolone acetate 1% qid, tapered each week over one month.

## 3. Results

**Figure 1:**
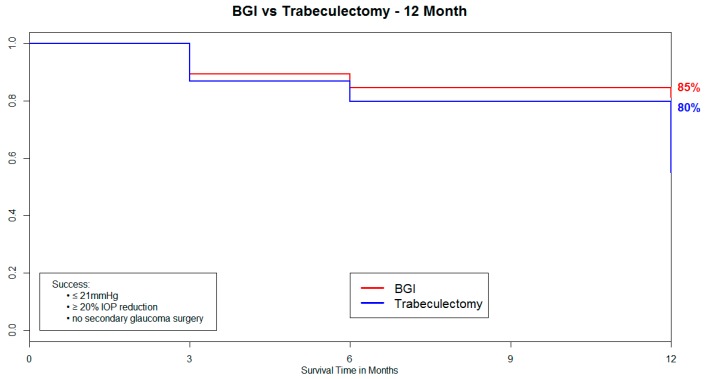
Survival curves of two groups.

**Figure 2:**
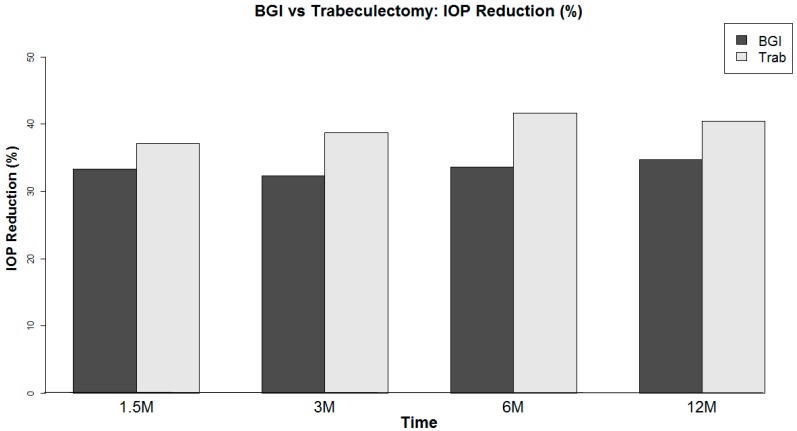
Percentage of IOP reduction.

A total of 57 eyes that underwent Baerveldt 101-350 glaucoma implant and 38 eyes that underwent trabeculectomy cases with ExPress™ shunt were included in this study (Table 1). In both groups, the primary diagnosis was primary open angle glaucoma. Kaplan–Meier analysis resulted in a survival rate at 12 months of 85% in the eyes that underwent Baerveldt glaucoma implant and 80% in the eyes that underwent trabeculectomy (Figure 1). No statistically significant difference was found in survival distributions between surgery groups using a log–rank test (Figure 1). A total of 12 trabeculectomy and 9 Baerveldt glaucoma implant cases failed by our definition of success. Of these failures, all 12 trabeculectomy with the ExPress shunt required further glaucoma surgery, and 5 of the 9 Baerveldt glaucoma implant cases required further glaucoma surgery. There was no case of endophthalmitis. These cases were included in the analysis of IOP, number of glaucoma medications, and visual acuity (VA). At one year follow-up, the mean IOP was reduced from 20.6 ± 5.6 mmHg to 12.4 ± 3.2 mmHg in the Baerveldt glaucoma implant group and from 20.7 ± 5.5 mmHg to 11.3 ± 4.8 mmHg in the trabeculectomy with ExPress shunt group (Table 2). On average, Baerveldt glaucoma implant showed an IOP reduction of 7.7 ± 6.1 mmHg, while trabeculectomy showed 7.9 ± 5.2 mmHg IOP reduction at one year post-operation (Table 2, Figure 2). No statistically significant difference was found in IOP reduction between groups at any visit (Table 2).

**Figure 3:**
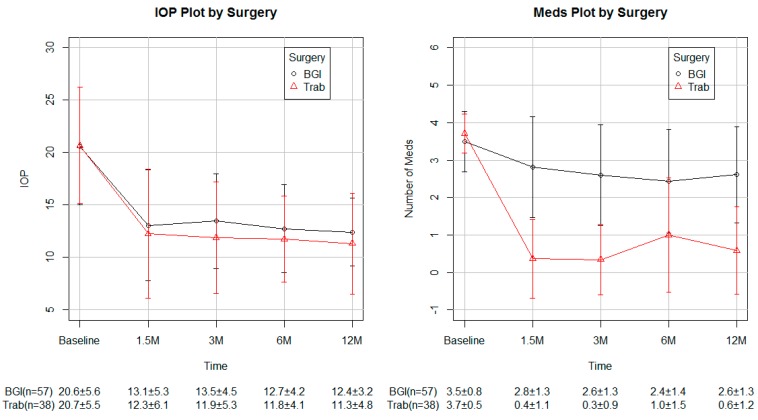
IOP and number of medications in each group (Mean ± SD).

Topical medications were reduced from 3.1 ± 1.0 to 2.5 ± 1.3 at one year in the Baerveldt glaucoma implant group and from 3.8 ± 0.4 to 0.3 ± 0.8 in the trabeculectomy group (Figure 3). The difference in medications used was statistically significant at all post-operative visits. At one year, the eyes that underwent the Baerveldt glaucoma implant had an average reduction of 0.8 ± 1.1 medications, while the eyes that underwent trabeculectomy had a reduction of 3.4 ± 1.1 (Table 3). Pre-operatively, the average Log mar was 0.5 ± 0.7 in the Baerveldt glaucoma implant group and 0.2 ± 0.3 in the trabeculectomy group (Table 4). At one year, the VA was 0.4 ± 0.4 in the Baerveldt glaucoma implant group, while the VA was 0.1 ± 0.1 in the trabeculectomy group (Table 4). A statistically significant difference was not found in the change of VA at any visit.

## 4. Discussion

This study is, to our knowledge, one of the largest investigations reviewing the success of Baerveldt glaucoma implantation versus trabeculectomy (with ExPress™ shunt) as a primary surgical treatment for primary open angle glaucoma. On average, both groups of eyes showed a similar level of IOP reduction (~10 mmHg) at one year post-operation. While the eyes that underwent trabeculectomy with ExPress shunt were less dependent on drops when successful, the Kaplan–Meier analysis demonstrated that the eyes that underwent glaucoma implant surgery were more likely to meet the criteria for success at one year. As a secondary measure, the VA was not very different in the two groups in post-operative follow-up.

The TVT trial was a landmark study that demonstrated similar outcomes up to five years post-operation when these two surgical modalities were used in the treatment of refractory glaucoma. The study, however, failed to address the potential difference in efficacy in eyes that are naïve to prior incisional glaucoma surgery, included eyes with very different glaucomatous diseases (neovascular glaucoma, uveitic glaucoma, and angle closure) compared to the most common open angle varieties of glaucoma and, given the nature of a large multicenter study with numerous surgeons, was unable to control for surgical techniques and post-operative management. At the time of the TVT trial, the ExPress™ device was not commonly used as an adjunctive device to trabeculectomy. This review was designed with these limitations in mind.

Panarelli et al. [5] investigated the efficacy of these two techniques as a primary surgical treatment for glaucoma. However, this retrospective review considered outcomes in a much broader variety of glaucomatous disease and encompassed a time period between 1985 and the present. The variety of glaucomatous disease does not fully address the efficacy of these surgical treatments in a cohort excluding non-open angle varieties of glaucoma. Additionally, given the time period over which these cases were reviewed, surgical techniques have evolved, and many surgeons modified their initial surgical approach to Baerveldt glaucoma implantation after its introduction in 1992 to achieve better outcomes. The ExPress™ shunt was also not in use during the majority of the period reviewed by these authors, and the preferred application methods of anti-metabolites are still evolving.

The methods of this study are unique in the standardized surgical techniques of the two surgeons, the use of the ExPress shunt as an adjunctive device with trabeculectomy, the standardized concentration and exposure of anti-metabolite Mitosol sponges (0.4 mg/mL Mitomycin C), and the consistency in the post-operative management. The exclusion of non-primary open angle glaucoma in this study is also different from previous investigations seeking to compare outcomes of glaucoma implants and trabeculectomy [3,5]. There are multiple factors to take into consideration in the initial choice of surgical modality for the treatment of primary open angle glaucoma. If medication intolerance, adherence, or instillation is a long-term obstacle to successful treatment, perhaps trabeculectomy may be a more suitable choice as this data suggests a much greater likelihood of achieving successful IOP reductions with much fewer medications. In addition, another factor that has traditionally led surgeons to choose trabeculectomy as an initial intervention is that if a primary glaucoma implant surgery were to fail and a future surgery is indicated, trabeculectomy is not likely to be successful.

The results of this review suggest that in eyes with open angle varieties of glaucoma naïve to incisional glaucoma surgery, both trabeculectomy and Baerveldt glaucoma implant are suitable choices to achieve a successful outcome with perhaps a higher rate of success in those eyes that underwent Baerveldt glaucoma implant. Most glaucoma surgeons would agree that appropriate follow-up intervention and care is key to the success of trabeculectomy. There are many practical considerations that may limit the ability to achieve a successful outcome in a patient who is otherwise a suitable candidate for a primary glaucoma surgery with trabeculectomy, such as an inability to follow-up due to distance, inability to instill a high frequency of steroid drops, poor access to the surgical site due to small palpebral fissure, contact lens use, and affinity for swimming. In these patients, with the understanding that it is likely that pre-operative drop medications would likely continue to be necessary, glaucoma implant surgery may be able to achieve a higher level of success than trabeculectomy. The definition of success is used as in prior investigations (TVT)—as a reduction in IOP of 20% from baseline and IOP < 21 and > 5 at all follow-up appointments.

The limitations of this study are similar to most retrospective reviews in that there is an inherent bias in the selection of patients for each modality, as patients who are in frail health or less able to follow up may be more likely to undergo Baerveldt glaucoma implant surgery. Furthermore, the majority of patients undergoing trabeculectomy also received the ExPress™ shunt instead of a traditional fistulization with a Kelly Descement punch. As with any study, using a standardized definition of success, success in an individual patient varies depending on the stage of disease, baseline IOP, and ability to use topical medication. Although these results suggest similar success with both surgical modalities, in patients with end stage glaucoma and an IOP goal of less than 12, and without other practical limitations, the most appropriate surgical modality may still be trabeculectomy. The absolute value of final IOP was still lower in the trabeculectomy group versus the Baerveldt glaucoma implant group (9.8 ± 3.4 mmHg versus 12.0 ± 3.3 mmHg). Further studies are needed to best define when each modality may be the better choice amongst patient with specific IOP targets.

## 5. Conclusions

The Baerveldt 101-350 glaucoma implant and trabeculectomy (with ExPress™) may have similar rates of success in the surgical treatment of primary open angle glaucoma in eyes that are naïve to prior incisional glaucoma surgery. Patients undergoing Baerveldt glaucoma implant surgery may have a slightly higher rate of success at one-year follow-up, but the patients undergoing trabeculectomy have a lesser need for topical medications.

## Figures and Tables

**Table 1:** Background metrics of patients undergoing Baerveldt 101-350 glaucoma implant (BGI) and trabeculectomy.

Baseline Metric	BGI (*n* = 57)	Trabeculectomy with Express (*n* = 38)
Intraocular Pressure (IOP)	20.6 ± 5.6	20.7 ± 5.5
Drop Medications	3.5 ± 0.8	3.7 ± 0.5
Visual Acuity (VA) (logMar)	0.5 ± 0.7	0.2 ± 0.3
Pseudophakia	1	4

**Table 2:** Intraocular pressure (IOP) and IOP reduction.

Follow up Time in Months (M)	BGI (*n* = 57)		Trabeculectomy with Express (*n* = 38)		*p*-Value
	IOP	Reduction in IOP	IOP	Reduction in IOP	
Baseline	20.6 ± 5.6	---	20.7 ± 5.5	---	---
1.5 M	13.1 ± 5.3	7.6 ± 7.0	12.3 ± 6.1	8.4 ± 8.0	0.36
3 M	13.5 ± 4.5	7.1 ± 5.7	11.9 ± 5.3	8.5 ± 7.3	0.22
6 M	12.7 ± 4.2	7.5 ± 6.3	11.8 ± 4.1	9.3 ± 6.3	0.33
12 M	12.4 ± 3.2	7.7 ± 6.1	11.3 ± 4.8	7.9 ± 5.2	0.75

**Table 3:** Drops and reduction in drops.

Follow up Time in Months (M)	BGI		Trabeculectomy with ExPress		*p*-Value (DRx)
	Rx	Reduction in Drops	Rx	Reduction in Drops	
Baseline	3.5 ± 0.8	---	3.7 ± 0.5	---	---
1.5 M	2.8 ± 1.3	0.7 ± 1.2	0.4 ± 1.1	3.3 ± 1.2	< 0.01
3 M	2.6 ± 1.3	0.9 ± 1.4	0.3 ± 0.9	3.4 ± 1.0	< 0.01
6 M	2.4 ± 1.4	1.0 ± 1.4	1.0 ± 1.5	2.8 ± 1.7	< 0.01
12 M	2.6 ± 1.3	0.9 ± 1.1	0.6 ± 1.8	3.2 ± 1.3	< 0.01

**Table 4:** Visual acuity (VA) and change in VA.

Follow up Time in Months (M)	BGI		Trabeculectomy with ExPress		*p*-Value
	logMar	Change in logMar	logMar	Change in LogMar	
Baseline	0.5 ± 0.7	---	0.2 ± 0.3	---	---
1.5 M	0.6 ± 0.6	−0.0 ± 0.4	0.3 ± 0.4	−0.1 ± 0.2	0.80
3 M	0.6 ± 0.6	−0.1 ± 0.4	0.2 ± 0.3	−0.0 ± 0.2	0.29
6 M	0.5 ± 0.7	−0.1 ± 0.5	0.2 ± 0.4	−0.0 ± 0.3	0.08
12 M	0.4 ± 0.4	−0.0 ± 0.2	0.1 ± 0.2	−0.0 ± 0.1	0.47
